# How to quit cannabis when you have a mental illness: study from the perspective of patients who have successfully quit

**DOI:** 10.1192/bjb.2023.69

**Published:** 2024-08

**Authors:** Jojanneke Bruins, Stijn Crutzen, Wim Veling, Stynke Castelein

**Affiliations:** 1 Lentis Psychiatric Institute, Groningen, The Netherlands; 2 University of Groningen, Groningen, The Netherlands

**Keywords:** Comorbidity, substance use disorders, qualitative research, severe mental illness, marijuana

## Abstract

**Aims and method:**

Research regarding quitting cannabis use often excludes patients with severe mental illness (SMI). We investigated facilitating and impeding factors in SMI patients and their advice to others, using semi-structured interviews with 12 SMI-patients, who were daily cannabis users for ≥12 months and had fully stopped using for ≥6 months.

**Results:**

Seeking distraction, social contacts in personal environment, avoiding temptation and support from professionals were facilitating factors in stopping. Impeding factors were withdrawal symptoms, user environment, experiencing stress and user's routine. Advice to other patients included to just do it, seek support from others, quit ‘cold turkey’ and acknowledge that cannabis use is a problem. Advice to mental health professionals is to discuss cannabis use from the start of treatment.

**Clinical implications:**

It is important to inform patients that cannabis use has negative consequences and limits the effects of treatment. Do not judge cannabis use or force the patient to stop.

There is a high prevalence of cannabis use among people with a severe mental illness (SMI).^1^ Cannabis use has been associated with many negative outcomes in people with SMI.^2–8^ Research on how to stop using cannabis in people with SMI is mostly limited to small studies with 16 to 90 participants.^9–11^ To date, the effects of (switching to) different medication have been reported to be non-existent or studies were underpowered.^9,12,13^ Similarly, psychosocial interventions such as motivational interviewing, cognitive–behavioural therapy and contingency management have only shown small, short-term effects^14^ or no effect at all in people with SMI.^15,16^ Technology-supported treatment shows minimal effects, but it is still in its early stages.^17^ Furthermore, as SMI diagnoses are often an exclusion criterion in addiction studies and the underlying motives for cannabis use in people with SMI^18–21^ appear to differ from those of the general population,^22^ findings from research in the general population cannot easily be generalised to the SMI population. It is therefore important to research successful quitting strategies in people with SMI. In this study, we conducted semi-structured interviews with people with SMI who had successfully stopped using cannabis for at least the past 6 months. We aimed to identify important facilitating and impeding factors from their experiences during the stopping process. Furthermore, we asked what advice they had for current SMI patients who wish to stop using cannabis and for mental health professionals who wish to help their patients quit.

## Method

### Study design and participants

Participants were recruited through purposive sampling in several psychiatric facilities in the northern Netherlands between September 2020 and July 2022. Patients were eligible for participation when they: (a) had a DSM-5 diagnosis of an SMI (psychotic, mood, anxiety, personality or post-traumatic stress disorder); (b) had used ≥3 units of cannabis per week for at least 1 year since their diagnosis, to exclude solely recreational users; and (c) had now successfully stopped using cannabis for at least 6 months, with no maximum discontinuation duration. The minimum threshold of 6 months was chosen because most cannabis interventions in people with SMI only show effects for 3–6 months.^23^ Written informed consent for audio recording of the interview and use of anonymised quotes was obtained from all participants prior to the interview. Audio files have been stored and encrypted and can only be accessed by the primary researcher (J.B.).

### Interview

We used a semi-structured questionnaire as the basis for the interview. Participants were asked open-ended questions and prompts were only provided if no answer was given or the conversation stagnated. The questions were categorised into three different aims. First (aim 1), we inventoried participants’ cannabis history by asking them to disclose details of their previous cannabis use, such as their frequency of use, reasons for use and whether they had attempted to quit before their successful attempt. Second (aim 2), we aimed to examine the stopping process of their successful attempt: their motivation to quit, which approach they had used to quit, which factors were helpful and which factors impeded the stopping process. Our final aim was to collect advice (aim 3); therefore, we asked the participants to share their advice for people in a similar situation to theirs, having both an SMI and cannabis addiction, and their advice for mental healthcare professionals who work with these patients. Interviews lasted approximately 30 min and were held by researchers with no relation to the participants, personally or professionally. Owing to the COVID-19 pandemic, most interviews were performed through video calls. We used the consolidated criteria for reporting qualitative research (COREQ) as a basis for our study design and report.^24^

### Analysis

Audio files were transcribed verbatim, and personal information was redacted (i.e. names, locations). We used Atlas.ti 22 for an inductive thematic analysis approach to identify themes within the three general areas of investigation. First, codes were generated from literal phrases in the transcripts and combined into transcended code groups. All transcripts were recoded using this code book. Subsequently, we summarised all code groups into themes. All transcripts were coded by at least two researchers. Disagreements were discussed with a third coder until consensus was reached. To validate our findings, we used investigator triangulation within the team that was involved in the interviews and coding, and peer debriefing with researchers and clinicians outside the main research team, as well as a member check with several of the participants.^25^

## Results

### Sample

A heterogeneous group of 12 participants was included in this study (R1–R12), aged from 22 to 45 years, of which six individuals had multiple diagnoses. An overview of the sample is presented in Table 1. The equipment malfunctioned in one interview; we therefore have only partial information available about facilitating and/or impeding factors (aim 2) and none about advice to others (aim 3) from participant R6.

**Table 1:** Overview of sample characteristics

	*N*
Sex, *n* (male)	5
Current age	(range: 22–45 years)
18–25 years	2
26–35 years	7
>35 years	3
Diagnoses^a^	
Psychotic disorder	5
Mood disorder	3
Anxiety disorder	3
Post-traumatic stress disorder	4
Personality disorder	3
Autism spectrum disorder	1
Age at onset of cannabis use	(range: 11–21 years)
≤15 years	8
16–17 years	2
≥18 years	2
Duration of discontinuance	(range: 0.75–12 years)
<1 year	3
1–5 years	5
>5 years	4
Daily use prior to discontinuance, *n* (yes)	12
Previous attempt to discontinue, *n* (yes)	7
Previous use of other substances, *n* (yes)	9
Alcohol, *n* (yes)	4
Nicotine, *n* (yes)	4
Other drugs than cannabis, *n* (yes)	9

a. Six participants had multiple comorbid diagnoses. Primary diagnosis were psychosis spectrum (*n* = 5), mood (*n* = 3), post-traumatic stress (*n* = 1), autism spectrum (*n* = 1) and personality (*n* = 2) disorders.

#### Cannabis use details

All participants were daily users of cannabis prior to stopping. Eleven participants had started for social reasons, being exposed to cannabis in their environment. One had started using to self-medicate against psychiatric symptoms.

##### Reasons for cannabis use

Most participants listed multiple reasons for having continued using while or after they had their diagnosis. The most frequently mentioned reason for continued use, given by all but one participant, was to help reduce their negative and undesired mental state (*n* = 11). Participants used cannabis to relieve stress, as sedation to numb themselves and/or to repress negative emotions.

‘*I noticed that I created calmness in my head with it. So yeah, at a certain point the reasons you are using become different. Then it's not because it is fun or social, but because you are like “Ah, I feel so nice and calm this way”.’ [R12]*

*‘I am great at numbing myself if I don't want to deal with something. Cannabis works great for that.’ [R2]*

The second reason was to self-medicate against psychiatric symptoms and problems related to sleeping (*n* = 8).

‘*It provides a sort of service, because you notice … Ok, I can easier … I have more control over my thoughts. And then it becomes very functional.’ [R1]*

*‘It made me sleep very well. A lot better than usual. But eventually you stop doing anything.’ [R3]*

Participants also reported using cannabis had become a routine, which resulted in a vicious circle (*n* = 7).

‘*I thought I was happy then, when I had used. Then when it wore off, I thought damn, shitty life, so I went and did it again.’ [R4]*

Half the participants (*n* = 6) also reported having continued using cannabis to experience its pleasant effects (i.e. feeling nice, creative), but none used cannabis solely for its pleasant effects.

#### Quitting process

##### Motivation to stop

The most frequently mentioned motivation was a rock-bottom moment (*n* = 8). A specific incident, or the realisation that their lives lacked meaning and future prospects, caused a turning point in their lives and motivated them to stop using.

‘*There was no more sense of day and night, but just the living hell. I had nowhere to go and that is what made me make the decision to enter the programme and check it out and give it a chance. But it was with the conviction “I don't want to live like this anymore”. So, I was really desperate.’ [R5]*

*‘I fell into a coma in 2009. It was a combination of drugs and alcohol and then I was in a coma for two weeks. That terrified me so much that I quit everything all at once.’ [R8]*

Half of the patients (*n* = 6) quit because of the practical consequences of their cannabis use, including increased severity of symptoms, involuntary hospital admissions and financial consequences (because cannabis is costly).

‘*I was completely sick of it. I had little money, the risk of admission, risk for a psychosis … ’ [R1]*

Some participants were motivated to stop because of others (*n* = 5), because they wanted to be a better partner (*n* = 2) or a responsible parent (*n* = 2) or pet owner (*n* = 1). It is important to note here that the participants explicitly mentioned that they were not convinced by others to quit, but that wanting to be there for others contributed to their intrinsic motivation to quit.

‘*The only reason I am not using cannabis anymore, I can be very honest about that, is my daughter.’ [R2]*

*‘I thought about my dog. I thought: “If I get admitted, where is he supposed to go?”.’ [R8]*

##### How to quit

All but one participant (*n* = 11) used a cold turkey approach to stop using cannabis, meaning that they stopped using at once. Two participants reported they were able to quit without help from others. Seven participants had professional help from mental health or addiction care workers or Narcotics Anonymous.

*[About mental healthcare worker] ‘When I quit, he came by often, called me all the time, sent me WhatsApp messages all the time. He really got me through it, yes.’ [R2]*

Four participants had help from people in their social network system and/or changed their personal environment. This included moving back in with their parents (*n* = 1), support from a new partner (*n* = 2), and going on holiday with family (*n* = 1).

‘*I went on holiday for a week with my family, so I had my mother and my brother close, so that I knew if I get annoying, they can take it and they can handle and support me. When I got back from holiday after that week, I stopped myself once or twice from going back to the coffeeshop again. Since then, I haven't needed it anymore.’ [R7]*

##### Facilitating factors

An overview of facilitating and impeding factors is provided in Box 1. The first most frequently mentioned facilitator was seeking distraction (*n* = 7). Participants disclosed that it helped them to undertake concrete activities that kept their minds occupied, such as exercising, biking, yoga, working, gaming, juggling, meditating or making music.

‘*Playing guitar, painting, playing darts. In the beginning I went juggling sometimes. When you start juggling you are only focused on those balls, you can't think about anything else. So that was helpful while kicking the habit.’ [R4]*

*‘Back then I adopted a dog and therefore had to go outside with that dog every day and such things. If I hadn't adopted that dog and had I not looked for a job, then I would now probably be … well … have withered at home or something.’ [R7]*

Box 1 Factors facilitating and impeding stopping cannabis

**Table:** 

Facilitating factors	
Seeking distraction Social contacts in personal environment Avoiding temptation Seeking support of professionals
Impeding factors Symptoms of withdrawal User environment Experiencing stress and negative emotions Cannabis as part of routine

The second facilitating factor was social contacts within their personal environment (*n* = 7), mainly receiving support from family and friends.

‘*New friends, who thought it was cool that I quit, that does help. When you have those friends who call you a loser when you have quit, it is nicer when you have friends who say: “Oh cool, great that you quit”.’ [R4]*

Another facilitator was avoiding temptation (*n* = 5). Participants got rid of cannabis paraphernalia and avoided contact with other users, so they would be less tempted to use again. Some went fully abstinent, because alcohol, for example, lowered the threshold to start using cannabis again.

‘*Look if I drink three, four, five beers or something, then at a certain point the standard is very low. That is what alcohol does and I am afraid that I will just take a detour to the [coffee] shop to get a joint.’ [R1]*

Finally, five participants named the support of professionals working in mental health and/or addiction care (*n* = 3) and peer support (*n* = 2) as facilitating factors in staying sober.

‘*No matter how willing people in your environment are to help you, if they don't get it from the inside out they will always say the wrong things. And as an addict everything you can use as a reason to start using again is a nice bonus. Someone only has to say the wrong word and you are like: “See, I am allowed!” You will use anything to be allowed to do it again. And they teach you to unlearn this habit here, when you are among peers. They will never give you that space.’ [R5]*

##### Impeding factors

Most participants reported that they went through a withdrawal phase when they stopped (*n* = 10). The most frequently mentioned symptoms of withdrawal were sleeping problems (e.g. nightmares, irregular sleep; *n* = 8), sweating (*n* = 6), eating problems (*n* = 2), tremors (*n* = 2) and (*n* = 1) physical pain. Seven of these participants reported these withdrawal symptoms as an impeding factor in their cannabis stopping process (see also Box 1).

‘*The sleeping, having to feel things again, that makes it hard to quit. And lots of nightmares, lots of sweating, at least for the first two weeks. After that it gets better. Withdrawal symptoms make it a lot harder to not start again.’ [R2]*

Second, half the participants also mentioned that living in a user environment made it difficult to maintain their sobriety (*n* = 6), for instance, having friends who were still using cannabis, easy access to cannabis, and cannabis use being trivialised and socially acceptable.

‘*I live in the heart of Amsterdam so I can walk in any direction and there will be a coffeeshop. And what also makes it difficult is that it is being downplayed so much.’ [R5]*

*‘My friends were all smoking cannabis and then they thought I was boring.’ [R8]*

Third, most participants used cannabis to suppress negative feelings and numb emotions. By quitting, they had to experience and cope with the full intensity of their (negative) emotions and stress again, which half of the participants (*n* = 6) said made it difficult not to start using again.

*‘The moment you quit you have that agitation back. That makes it hard.’ [R2]*

*‘I had to be careful with relationships. Careful with entering relationships, intense negative emotions, which are involved in relations. They can sometimes cause cravings, that you wish to easily suppress those feelings.’ [R9]*

Finally, cannabis simply being part of their routine made it difficult for some (*n* = 4) to kick the habit and stay sober. Notably, one participant mentioned it was annoying when professionals asked about the number of joints they smoked instead of the quantity of cannabis content they used, which they said was much more relevant.

#### Advice to others

##### Advice to SMI patients who wish to stop using cannabis (Box 2)

The advice most frequently given by our participants was that intrinsic motivation is needed to truly get behind a stopping attempt (*n* = 7). To get your mindset right and not give up, because it is a matter of wanting to do it and not being able to do it.

‘*Get completely behind it. Get your mindset ready and then just do it. You have to be 100% behind it, otherwise it won't work. If it fails once, just try again.’ [R4]*

*‘There is always a reason to continue, because now is not the right time, because you think: “I don't want to stop right now”. But you can get off it. On the one hand your brain locked you up, so you are going back in circles. But it is your brain, so your brain can also make a new key to get out of that.’ [R1]*

Box 2 Overview of the advice given by participants to other SMI patients and mental health professionals

**Table:** 

Advice to SMI patients	
Get your mindset right and just do it Seek help and support from others Quit ‘cold turkey’ Acknowledge cannabis as an addiction and a problem
Advice to mental health professionals Discuss cannabis use right at the start of treatment. No judging, persuasion or forcing them to stop, but provide neutral information Connect as a human being and make your help easy and accessible Promote active therapy

They also advised seeking help and support from others (*n* = 6). Participants indicated that, especially in the beginning, seeking professional help is beneficial to your stopping attempt, whether it is in mental healthcare, addiction care or a support group such as Narcotics Anonymous.

‘*Don't try to fix it alone purely on willpower. Thinking: “I'm going to throw everything out and then I am going to sit alone in my house, hoping that tonight I don't get the urge to … ”. You cannot do this alone.’ [R5]*

Four participants also pointed out that they believe it is best to quit all at once, or ‘cold turkey’ as they say, and to not be too hard on yourself.

‘*I don't think phasing out works with [cannabis]. I think the best option is to just quit all at once.’ [R2]*

Some (*n* = 3) advised that acknowledging your cannabis use as an addiction and recognising that it is indeed a problem is an important part of being able to stop and to stay off cannabis.

‘*Everyone around you says it is no problem, it is not that bad and it is only a joint. But this is truly a serious problem and that changes your mindset. It changes everything, so just take it seriously as an actual problem.’ [R5]*

*‘I think about it every day. I think I have successfully stopped, but I don't think I will ever lose my addiction. It is always in my head.’ [R1]*

#### Advice to professionals working in mental healthcare

Participants advised treating the mental illness and cannabis addiction simultaneously by making cannabis use discussable with patients right from the start (*n* = 7). It is important not to judge or try to convince them to stop, but only to inform them.

‘*If I had understood my addiction sooner, it might have saved me ten years of treatment. So if you have someone with mental health problems who is also addicted [to cannabis] and you treat them both at the same time, so this and that … I think you get more efficiency from treatment when people stop using. It would have made a big difference for me.’ [R1]*

*‘Say: “Therapy works better if you quit”, but not: “You have to!”, and not: “I have a lot of opinions about that”.’ [R5]*

Participants also recommended connecting with patients on a human level (*n* = 5): be understanding and approachable and create a safe environment, to make the threshold for seeking your help low and make all help easily accessible.

‘*Of course, they noted the cannabis use every time. They did not stimulate it of course, like, go ahead and smoke or whatever, but they showed understanding for it and I really appreciated that.’ [R12]*

*‘You are addicted to something, so it's not going to go right all at once. I think a mental aid worker should respond to that, just like … You shouldn't tell someone: “You shouldn't have done that”, et cetera … He should say: “We'll try again next time”.’ [R10]*

*‘The feeling that you don't have to do it alone, that you don't have to be ashamed about it. Also that the threshold is low [.] and that there is always a safety net for you.’ [R5]*

Participants also recommended that professionals should promote activity, for example, active therapy such as yoga, creative therapy or psychomotor therapy, or encourage patients to undertake pleasant activities by themselves.

‘*Do some gardening with your patients or make a painting with them. Or do yoga during your [treatment] session with them. Or at least do something else than just talk about it more.’ [R5]*

Two other participants noted that mental healthcare workers could help patients get through the first weeks by providing medication for withdrawal symptoms and/or offering additional therapy sessions during the initial phase of the stopping process.

## Discussion

In this study, we conducted 12 interviews with SMI patients who had successfully stopped using cannabis. The main reasons for their previous cannabis use were to help reduce a negative and undesired mental state and to self-medicate. Their quitting experiences offered several concrete factors that may be useful for future SMI patients and their mental healthcare workers to help them stop using cannabis. They named intrinsic motivation as a necessary requisite for being able to stop, with the addendum that intrinsic motivation can build over time and that others could contribute to this process. Seeking help and quitting at once (cold turkey) were the most commonly used strategies that led to successful discontinuation, but otherwise there was no one-size-fits-all method to stop using cannabis. Important factors in general were avoiding temptation and seeking distraction, seeking support from professionals (including peer support) and the patient's personal environment. The most important factors that impeded the stopping process were a cannabis-friendly environment, going through withdrawal and having to fully experience negative emotions and agitation without the numbing effects of cannabis.

Although the initial reasons participants named for starting to use cannabis (social reasons and exposure from their environment) were comparable with those of the general population,^22^ participants in this study named reducing undesired mental states (i.e. feeling calm and suppressing negative emotions) and self-medication as the main reasons for their continued use, similar to the findings of previous studies about motivation for cannabis use in people with an SMI.^19,21^ They also emphasised that intrinsic motivation is an essential element of the stopping process, which is in line with existing research regarding cannabis addiction in the general population.^26^ Although quitting cannabis often does not last when people stop *for* others, others can be(come) a factor that contributes to building intrinsic motivation. This further emphasises the importance of motivational interviewing during therapy to build patients’ intrinsic motivation to stop using.^27^ It also calls into question the long-term usefulness of contingency management interventions,^28^ which are based on external motivation, where people receive rewards for their abstinence, although these might still be useful to achieve important harm reduction in the short term. Using motivational interviewing to build intrinsic motivation to stop using would also be in line with the general treatment recommendation to use an integrated approach where both psychosis and cannabis use are treated simultaneously.^29–31^ This was also given as concrete advice by our participants to mental health professionals. Only a minority actively mentioned their mental healthcare worker as a helpful factor during their stopping attempt, indicating that the quality and/or quantity of support from mental healthcare workers could be improved. Furthermore, the small short-term effects of motivational interviewing on quitting cannabis in the SMI population suggest that once the intrinsic motivation is sufficiently present, motivational interviewing might not be enough to actually help these patients to stop using.^15,16^

The Dutch guidelines recommend involving a patient's system during a stopping attempt.^31^ This is in line with the findings of our study, where personal environment was reported as a facilitating factor in stopping cannabis. The same guidelines also recommend a phasing-out schedule,^30^ although our findings suggest that patients should stop at once. Unfortunately, most (inter)national SMI treatment guidelines only recommend referring to secondary/addiction care,^32,33^ comment that there is no sufficient scientific evidence to make recommendations ^34^ and/or refer to general addiction guidelines, which are generally based on research that excludes people with SMI.^33,35^

Notably, many participants described how their social contacts had changed in relation to quitting cannabis, either by choice or as a consequence. Some deliberately chose to avoid certain contacts or social environments in order to not be tempted to start using again. For others, it was a consequence of quitting cannabis, because they simply did not run in the same circles anymore.

### Strengths and limitations

To our knowledge, this is the first study to examine factors that have facilitated and impeded the cannabis stopping process in SMI patients who have successfully stopped using cannabis. Given their long duration of (daily) use, the participants in our study sample were able to provide advice and insight into factors facilitating the stopping process. The findings send a hopeful message that even serious cannabis users with multiple addictions and comorbidities are able to stop using. It is possible that by only including success stories, we have missed some impeding factors. However, 58% of our sample had unsuccessfully tried to stop on a previous occasion and referred to these experiences in their interviews as well. Furthermore, we did not record whether patients were still using other substances, although four participants (R1, R5, R6 and R8) spontaneously mentioned being fully abstinent. Information regarding prescribed medication and the method of cannabis use (e.g. with or without tobacco) was not systematically collected but would have been helpful in the interpretation of our findings, as certain withdrawal experiences might not be exclusively cannabis-related. Finally, study results were reported in accordance with the COREQ criteria for qualitative research.^24^

### Clinical implications for SMI patients

Patients should acknowledge that using cannabis is a problem and an addiction. When they are ready to quit, they should consider stopping at once and seek both professional help and support from their personal environment. It is recommended that they temporarily leave their own environment during the first weeks, for example, by going to an addiction clinic or on a holiday with others who can help support them through withdrawal. Disposing of cannabis paraphernalia, avoiding (social) situations with other users and seeking distraction to keep their minds occupied may be helpful.

### Clinical implications for clinicians

Mental illness and cannabis use should be treated simultaneously, as recommended by the treatment guidelines, by discussing cannabis use openly right from the start.^29–31^ Judging and forcing a patient to quit will not help in the long term. Instead, provide factual information about the negative impact of cannabis use and inform the patient that any treatment will not reach its maximum potential effect while they continue to use cannabis. Emphasise that you would advise them to stop, but that it is their decision and that they do not need to hide it. Subsequently, use motivational interviewing techniques, where you discuss patients’ motivations for using, listen with empathy and empower them,^27^ to help build their intrinsic motivation to stop using cannabis and keep the threshold for seeking your help low. Once patients are motivated to quit, there is no one-size-fits-all approach. Quitting at once may be preferable compared with using a phasing-out schedule. Increasing the frequency of therapy sessions (temporarily) is advised to help the patients stay motivated and enable you to monitor the difficulties together, which you can then address during therapy. The most important thing to remind patients of is that they should try again if they do not succeed at the first try.

## Data Availability

The data that support the findings of this study are available on request from the corresponding author, J.B. The data are not publicly available owing to the personal nature of the interviews, which could compromise the privacy of research participants.

## About the authors

**Jojanneke Bruins**, PhD, is a senior researcher at Lentis Psychiatric Institute, Lentis Research, Groningen, The Netherlands; and University Medical Center Groningen, University Center for Psychiatry, Rob Giel Research Center, University of Groningen, Groningen, The Netherlands. **Stijn Crutzen**, PhD, is a senior researcher at Lentis Psychiatric Institute, Lentis Research, Groningen, The Netherlands; and University Medical Center Groningen, University Center for Psychiatry, Rob Giel Research Center, University of Groningen, Groningen, The Netherlands. **Wim Veling**, MD, PhD, is a professor of psychiatry at University Medical Center Groningen, University Center for Psychiatry, Rob Giel Research Center, University of Groningen, Groningen, The Netherlands. **Stynke Castelein**, PhD, is a department head at Lentis Psychiatric Institute, Lentis Research, Groningen, The Netherlands; and professor of recovery in severe mental illness at the Faculty of Behavioral and Social Sciences, University Medical Center Groningen, University Center for Psychiatry, Rob Giel Research Center, University of Groningen, Groningen, The Netherlands.
