# How to quit cannabis when you have a mental illness. A study from the perspective of patients who have successfully quit – ERRATUM

**DOI:** 10.1192/bjb.2023.87

**Published:** 2024-08

**Authors:** Jojanneke Bruins, Stijn Crutzen, Wim Veling, Stynke Castelein

This article was originally published with an incorrect affiliation for Wim Veling. The correct affiliation is University of Groningen, University Medical Center Groningen, University Center for Psychiatry, Groningen, The Netherlands.

The error has been corrected and the online PDF and HTML versions updated.
